# Assaying Silver Compounds in the Wet Way

**Published:** 1869-04

**Authors:** 


Assaying Silver Compounds in the Wet Way.—M. Stas makes the following remarks on this point: The mode of testing in the wet way in order to fix the standard of silver substances, as established by Gay-Lussac, is open, under certain conditions, to a source of error, arising from the solubility of chloride of silver in the very liquid to which its origin is due. This solution, whatever its mode of production may be, is precipitated equally by a decimal solution of silver and by chlorhydric acid. The extent to which this precipitation ensues is uncertain. At the ordinary temperature, there may be a variation from one to six thousandths in 100 c.c. of the liquid. Practically, it is quite possible, while still preserving the simplicity of the wet method, as invented by Gay-Lussac, to substitute a bromide for a chloride in precipitating silver, and thus remove absolutely those anomalies which have been observed to be attendant upon the use of a chloride or of chlorohydric acid.—*Vide Chemical News*, January 1.

